# Improving the Diagnosis of Bloodstream Infections: PCR Coupled with Mass Spectrometry

**DOI:** 10.1155/2014/501214

**Published:** 2014-04-09

**Authors:** Elena Jordana-Lluch, Montserrat Giménez, M. Dolores Quesada, Vicente Ausina, Elisa Martró

**Affiliations:** ^1^Servei de Microbiologia, Fundació Institut d'Investigació en Ciències de la Salut Germans Trias i Pujol, Hospital Universitari Germans Trias i Pujol, Universitat Autònoma de Barcelona, 08916 Badalona, Spain; ^2^CIBER Enfermedades Respiratorias (CIBERES), 07110 Bunyola, Spain; ^3^CIBER Epidemiología y Salud Pública (CIBERESP), 08036 Barcelona, Spain

## Abstract

The reference method for the diagnosis of bloodstream infections is blood culture followed by biochemical identification and antibiotic susceptibility testing of the isolated pathogen. This process requires 48 to 72 hours. The rapid administration of the most appropriate antimicrobial treatment is crucial for the survival of septic patients; therefore, a rapid method that enables diagnosis directly from analysis of a blood sample without culture is needed. A recently developed platform that couples broad-range PCR amplification of pathogen DNA with electrospray ionization mass spectrometry (PCR/ESI-MS) has the ability to identify virtually any microorganism from direct clinical specimens. To date, two clinical evaluations of the PCR/ESI-MS technology for the diagnosis of bloodstream infections from whole blood have been published. Here we discuss them and describe recent improvements that result in an enhanced sensitivity. Other commercially available assays for the molecular diagnosis of bloodstream infections from whole blood are also reviewed. The use of highly sensitive molecular diagnostic methods in combination with conventional procedures could substantially improve the management of septic patients.

## 1. Introduction

Bloodstream infection is a life-threatening condition that results from the presence of microorganisms, generally bacteria or fungi, in the blood [1]. The time window for the administration of an appropriate therapy is less than 6 hours once the symptoms are recognized, and it is optimal to administer broad-range antibiotics within the first hour, preferably after obtaining a blood culture for microbiological diagnosis [2]. Inadequate antimicrobial therapy increases the risk of mortality. Every hour of delay in initiation of appropriate antimicrobial therapy increases the mortality by 7.6% in patients with septic shock [3]. Conventional methods for the microbiological diagnosis of sepsis rely on blood culture followed by biochemical identification. It usually takes 1 to 3 days to obtain both the identification and the antimicrobial susceptibility profile of the pathogen. The major limitation of blood culture methods is that they require a median time-to-positivity of 12 to 17 hours [4]. Another limitation of this method is that the presence of unculturable or fastidious microorganisms may decrease its sensitivity. Culture may also be negative if antimicrobial therapy was begun prior to blood sampling. Thus, there is an urgent need to improve the diagnostic tools for a better management of septic patients.

The ideal diagnostic platform should identify a broad spectrum of pathogens (bacteria, fungi, viruses, and protozoa), determine the susceptibility to a battery of antibiotics, allow the analysis of specimens in high or low throughput, have a low cost per sample, have minimum hands-on time, be user friendly, and, ideally, generate the results in a timely manner for the management of septic patients (6 hours or less). Mass spectrometry technology has recently been introduced in the clinical microbiology laboratory. Using matrix-assisted laser ionization time-of-flight (MALDI-TOF) spectrometers the diagnostic process may be shortened [5–7] as the identification of the pathogen can be achieved within 30 minutes directly from a positive blood culture [8]. Additionally, this technology is able to detect the resistance to some antibiotics [9], such as the presence of *β*-lactamases (including carbapenemases), methicillin-resistant* Staphylococcus aureus,* and even vancomycin-resistant* Enterococcus* spp. However, this technology relies on culture and, therefore, a median of 12- to 17-hour delay is unavoidable [4]. In order to further accelerate the diagnostic process, it is desirable to detect and identity pathogens directly from the patient's blood, avoiding the culture step.

Several molecular methods have been developed for the detection of pathogens (mainly bacteria and some fungi) in whole blood. The first assays developed were designed for the detection of a single pathogen of interest and are reviewed by Klouche and Schröder [10]. A single-pathogen approach is not useful for the diagnosis of bloodstream infections, as these infections may be caused by a broad range of microorganisms. This limitation has been overcome in several commercial assays which are able to detect a number of microorganisms [10–12]. These assays are based on two main strategies: the identification of a selected group of pathogens using specific targets (i.e., SeptiFast [13], VYOO [14], and Magicplex [15]) or the detection of a broad range of pathogens using universal/conserved targets (i.e., SepsiTest [16], PCR/ESI-MS [17]).

Use of whole blood in assays designed to detect pathogen nucleic acid is challenging. An excess of human DNA may hamper the detection of pathogen genomic material or may inhibit the PCR reaction [18, 19]; hemoglobin traces may also inhibit PCR-based amplification. Therefore, molecular methods are forced to use a relatively small volume of blood (1 to 5 mL); whereas conventional culture methods use 20–30 mL. This limited volume reduces sensitivity of the molecular methods. Additionally, the bacterial load in adults with bloodstream infection can be as low as 1–10 CFU/mL [20], which may preclude detection of pathogen DNA.

The PCR/ESI-MS technology combines broad-range PCR amplification with the electrospray-ionization time-of-flight mass spectrometry, which is a highly sensitive detection method. Methods have now been developed that allow use of the PCR/ESI-MS technology on whole blood samples, and two clinical evaluations of this system have recently been published [21, 22]. A new version of the instrument is presented that has been designed to improve the sensitivity and implementation in the clinical laboratory. This review describes the current status of the molecular diagnosis of sepsis with emphasis on the PCR/ESI-MS technology.

## 2. Summary of Commercially Available Molecular Assays for the Diagnosis of Bloodstream Infections from Whole Blood

### 2.1. SeptiFast (Roche, Mannheim, Germany)

SeptiFast is a multiplex real-time PCR assay that detects 25 pathogens including five* Candida* species and* Aspergillus fumigatus *[13]. The presence of the resistance gene* mec*A may be detected with a separate test. The initial volume of blood required is 3 mL (using the manual DNA extraction protocol 1.5 mL aliquots are processed in duplicate) or 1.5 mL (using the automated DNA extraction) [23]. The region amplified in this assay is the internal transcribed spacer region (IST), which is located between the 16S and 23S ribosomal genes for bacteria and between 18S and 5.8S ribosomal genes for fungi [13]. The amplification is performed with a LightCycler 2.0 instrument; different pathogens are detected through specific fluorescent probes. The time-to-result using this approach is 4.5–6 hours. This assay has been widely evaluated in the clinical setting; however, the results are conflicting with reported sensitivities ranging from 15% to 98% in ICU patients [24]. Recently, Chang et al. reviewed all the available literature reporting use of the SeptiFast assay and performed a meta-analysis that included data on 6,012 patients from 35 selected studies. The overall calculated sensitivity of SeptiFast was 75.0% (95% confidence interval, 65.0–83.0%), and the specificity was 92.0% (95% confidence interval, 90.0–95.0%). The performance of the test clearly varies depending on the group of patients tested.

### 2.2. SepsiTest (Molzym, Bremen, Germany)

The SepsiTest assay is based on broad-range PCR amplification followed by sequencing. In the SepsiTest two 1 mL aliquots of blood are processed in duplicate and human DNA is selectively degraded prior to the bacterial cell lysis step [16]. Several studies using this approach for the diagnosis of sepsis have been published. The largest study (*N* = 342) [16] reported a sensitivity and specificity of 87.0% and 85.8%. Two smaller studies reported lower values of sensitivity of 46.0% (*N* = 50) [25] and 37.5% (*N* = 75) [26]; specificities were 100% [25] and 86.6% [26].

### 2.3. VYOO (SIRS-Lab, Jena, Germany)

The VYOO assay is a multiplexed PCR analysis that detects 34 pathogens, including six species of* Candida *and* Aspergillus fumigatus, *as well as several resistance genes (methicillin resistance gene* mec*A, vancomycin resistance genes* van*A and* van*B, and *β*-lactamase genes* bla*SHV and* bla*CTX-M). The amplified products are visualized using a conventional gel electrophoresis, and the time-to-result is 8 hours. For this assay, microbial DNA from 5 mL of blood is enriched: total DNA is applied to an affinity chromatographic column that specifically binds the microbial DNA (LOOXTER) [27]. Additionally, human DNA is depleted during the extraction step. This assay has a sensitivity ranging from 38.0% to 60.0% [14, 25, 28].

### 2.4. Magicplex Sepsis Real-Time Test (Seegene, Seoul, Korea)

In the Magicplex Sepsis assay, three PCR reactions are necessary to achieve the identification at the species level of the pathogen. First, a conventional PCR amplification step is performed. In this step, primers designed to amplify genomic material from 91 microorganisms (85 bacteria, five species of* Candida,* and* Aspergillus fumigatus*) and three resistance genes (methicillin resistance gene* mec*A and vancomycin resistance genes* van*A and* van*B) are used. A real-time PCR is then carried out in a screening step for identification of the group or genera level of pathogens present. Finally, a second real-time PCR is performed to achieve the identification at species level. Identification of 21 bacterial species, five* Candida* species, and* Aspergillus fumigatus* is possible. For the DNA extraction, 1 mL of whole blood is used and human DNA is removed prior to the lysis of microorganisms. The time-to-result of this assay is 6 hours. To our knowledge, only one study using this approach for the molecular diagnosis of sepsis has been published [15]. The sensitivity and specificity were reported to be 65.0% and 92.0%, respectively.

## 3. The PCR/ESI-MS Technology

### 3.1. Principles of the Technology

**Figure 1:**
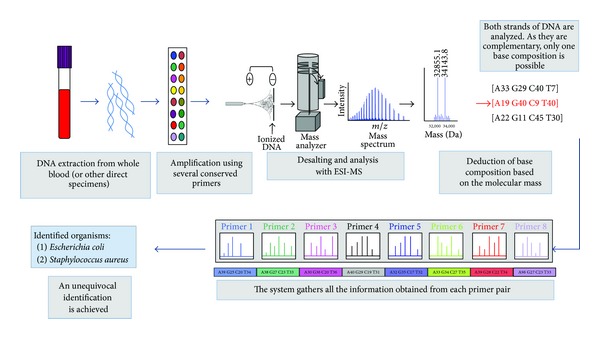
Schematic workflow of PCR/ESI-MS system. ESI-MS: electrospray ionization mass spectrometry. Part of the figure has been modified from Ibis Biosciences, a subsidiary of Abbott Molecular, with permission.

This technology combines broad-range PCR with ESI-MS mass spectrometry. Briefly, after the PCR, amplicons are desalted and analyzed by mass spectrometry. ESI-MS is used to determine the molecular mass of each amplicon, which is then used to calculate the base composition of each amplicon. The base compositions of multiple amplicons from different regions of the genome are compared to an extensive database and the identification of the pathogen is achieved (Figure 1). Even though the base composition analysis is not as informative as sequencing, it has enough discrimination power for the detection and identification of hundreds of microbial pathogens. A broad bacteria and* Candida* detection assay (BAC assay; Ibis Biosciences, an Abbott company, Carlsbad, CA, USA) has been designed for use in clinical research to identify more than 600 bacteria and* Candida* species. The BAC assay also detects resistance genes for three clinically relevant antibiotics: methicillin (*mec*A), vancomycin (*van*A and* van*B), and carbapenem (*blaKPC*).

### 3.2. PCR Amplification

The amplification of conserved regions of the genome has been widely used for the identification of microorganisms at the species level. Although the most common targets are the ribosomal DNA genes (i.e., 16S for bacteria and 18S for fungi), several housekeeping genes (i.e.,* tufB*,* rplB*,* valsS*, and* rpoB*) are also useful for the identification of pathogens [10, 11, 29]. Within these genes, highly conserved regions are used as priming sites, but the region amplified contains enough variability for the discrimination between species. For instance, in order to identify bacterial and* Candida* species, the BAC assay includes thirteen pairs of primers targeting different conserved regions (nine primers pairs for bacteria and four for* Candida* species). An advantage of using PCR primers designed for several conserved regions with varying degrees of specificity is that when more than one microorganism is present, there is redundancy of coverage across various primer pairs. This is especially relevant when the different microorganisms are present in different abundances, as using several nonoverlapping primer pairs may allow amplification of the less abundant species. Redundant amplification also prevents missed detections due to mismatches in single priming sites [29, 30].

### 3.3. Detection and Quantification of PCR Products

Mass spectrometry is highly sensitive and can detect small amounts of a nucleic acid of a given sequence even in a complex mixture. The PCR/ESI-MS system employs a software algorithm that calculates a base composition for each amplicon based on mass, compares these to an extensive database, and achieves the identification of the pathogen [17, 31].

Another feature of this technology is that it allows a relative quantification of the microorganism present in the specimen. This is achieved by the use of an internal standard that is amplified with the same primer pairs as those for amplification of the target gene. The internal standard has a different base composition and thus can be differentiated. As this synthetic standard is added to each PCR well at a known copy number, the comparison between standard and microbial DNA permits quantification. In the absence of a PCR product, the internal standard serves as PCR positive control to exclude PCR inhibition.

### 3.4. Usefulness of the PCR/ESI-MS for the Diagnosis of Bloodstream Infections

The accuracy of BAC assay for the diagnosis of bloodstream infections was first evaluated on blood culture specimens [32–34]. Those studies demonstrated robustness of the technology in terms of accuracy of the identifications. However, with the introduction of MALDI-TOF instruments for the identification of pathogens from positive blood culture based on their protein/peptide profile, it became clear that PCR/ESI-MS would not be able to compete on either a time-to-result or cost-per-sample basis with MALDI-TOF [35].

An advantage of the PCR/ESI-MS assay relative to the MALDI-TOF assay is that PCR/ESI-MS has been optimized to achieve a rapid diagnosis from direct clinical specimens. To date, two clinical evaluations of the PCR/ESI-MS for the diagnosis of bloodstream infections from whole blood have been published. Jordana-Lluch et al. [21] evaluated this system analyzing 247 whole blood specimens (75 with a paired positive blood culture and 172 with a negative blood culture result), and Laffler et al. [22] tested 464 whole blood specimens with a positive paired blood culture and 442 with a negative blood culture result. The agreement between blood culture followed by biochemical identification and PCR/ESI-MS was good in both studies: 77.1% in the Jordana-Lluch et al. study [21] and 78.6% in the Laffler et al. study [22].

Polymicrobial infections were detected in both studies by conventional and/or molecular methods. The agreement between methods on these specimens was low, as most of the mixed infections were detected by only one of the two methods. However, the use of this molecular method in addition to blood culture would have resulted in additional detections of clinically relevant microorganisms in some cases, which could have influenced patient outcome.

In a number of cases in both studies, PCR/ESI-MS detected microorganisms in whole blood specimens with a paired negative blood culture. The clinical relevance of the additionally detected microorganisms was investigated through clinical records review in order to discriminate between probable contaminants and true pathogens. The proportions of detected microorganisms with clinical significance not isolated by conventional methods were 7.5% (13 out 172 blood culture negative cases) [21] and 7.2% (31 out of 431 blood culture negative cases) [22]. These findings are highly relevant, as conventional methods were not able to diagnose the etiology of infection in the culture-negative patients.

The sensitivity of the system was calculated using different approaches in each study. Jordana-Lluch et al. disregarded those specimens with a polymicrobial identification by either or both methods, as the events with one correct detection but with a disagreement in the second one were difficult to catalogue as “true positive” or “false positive.” In those terms, the sensitivity of the PCR/ESI-MS was 50.0%. Laffler et al. performed a theoretical approximation of the sensitivity based on the historical blood culture positivity rate in their center. They extrapolated the experimentally obtained PCR/ESI-MS positivity rate in order to obtain the number of negative blood cultures that, if processed by the PCR/ESI-MS, would have additionally tested positive. The estimated sensitivity of PCR/ESI-MS using this theoretical approach was 85.9%. This extrapolation may have led to a biased estimation of the sensitivity.

Although these sensitivity values are not directly comparable because they were calculated in different ways, the Laffler et al. study had a higher detection rate of the PCR/ESI-MS on whole blood specimens with a paired positive blood culture. As many factors may affect the sensitivity of molecular methods, a direct comparison between studies is difficult. Differences in the clinical condition of the patients, their characteristics (e.g., age, antimicrobial treatment at the time of the blood draw), the microorganisms isolated, the number of blood cultures taken, and the volume of blood drawn for culture may result in differences between studies [36]. The limitations in sensitivity of the evaluated version of the PCR/ESI-MS technology result from the amount of blood tested in comparison with the blood culture (1.25 versus 20–30 mL). This problem has been overcome with the new version of the PCR/ESI-MS technology, which uses higher volumes of whole blood reducing the limit of detection 4-5-fold.

### 3.5. The New Version of PCR/ESI-MS

Since its original description by the team of Ibis Biosciences, the PCR/ESI-MS technology has been continuously evolving. The first instrument, named TIGER (for Triangulation Identification for the Genetic Evaluation of Risk) [31], was initially designed for biodefense and surveillance applications, due to its capability to identify previously unknown and unculturable microorganisms. Shortly after, a commercial version of this technology appeared, the Ibis T5000 [17, 30]. In this format, the sample processing was automated and a software system permitted management of the instrumentation, signal analysis, and report generation. This version of the instrument was intended to be used in health and industry settings; it provided highly sensitive detection without the need for a highly trained operator. With the incorporation of Ibis Biosciences into the Abbott group, the system was upgraded [29]. This system, the PLEX-ID, was used in the aforementioned studies [21, 22, 32, 33, 35]. Recently, a newer version has been developed with improvements focused on the analysis of direct patient specimens. One of the principal changes is the use of a larger volume of blood (5 mL) in order to increase sensitivity. Changes in the extraction process allow the use of several types of primary tubes and extraction protocols are tailored to the needs of the clinical laboratory. Another important improvement is that one to six specimens can be analyzed at a time. Finally, the mass spectrometer is a bench-top instrument, facilitating installation in clinical laboratories. In Table 1, a comparison between the PLEX-ID and the new version of the PCR/ESI-MS technology is depicted. A preliminary evaluation of this new version has shown a better sensitivity in the detection of pathogens in direct clinical specimens. Further evaluations are currently underway.

### 3.6. Other Applications in the Clinical Diagnosis of Infectious Diseases

The versatility of the PCR/ESI-MS has been widely demonstrated. In 2012, Wolk et al. [37] reviewed the existing literature of this technology. In this section, we aim to summarize its potential applications in the clinical laboratory as well as to point out several new publications not included in the previous review.

A PCR/ESI-MS assay is able to differentiate species in the* Mycobacterium tuberculosis* complex and classify these species based on drug resistance [38, 39]. This technology has also proved its usefulness for epidemiological proposes, given that it enables molecular genotyping [40]. For instance, genotyping of* Staphylococcus aureus *[41, 42],* Acinetobacter baumannii *[43–45], and respiratory pathogens [46, 47] has been performed in a variety of clinical settings. Bhatia et al. [48] used PCR/ESI-MS to identify a* Streptococcus intermedius* species from cerebrospinal fluid (CFS) and from a fixed biopsy in a patient with a central nervous system (CNS) infection. Although this infection had a respiratory origin, both bronchoalveolar lavage and CFS cultures were negative. Farrell et al. [49] investigated the capability of PCR/ESI-MS to identify pathogens on several specimens collected from patients undergoing antimicrobial treatment. A total of 76 clinical specimens including swabs, blood cultures, fluids, and tissues were collected from 47 patients. From those, 72% (55/76) were culture negative, whereas 76% (58/76) were PCR/ESI-MS positive.

Major viral families can also be detected using this approach. Of special interest is the new version of the Viral IC assay designed for the diagnosis of opportunistic viral infections of immunocompromised patients by viruses such as* Herpesvirus*,* Adenovirus*,* Parvovirus*,* Picornavirus*, and* Polyomavirus*. The ability of the assays on the PCR/ESI-MS system to detect influenza virus, coronavirus, respiratory syncytial virus, human adenovirus, human metapneumovirus, vector-borne flaviviruses, and alphaviruses has been demonstrated [50–52]. Moreover, this technology shows a great promise for the global surveillance of influenza virus [53–55]. Remarkably, it was able to detect the novel H1N1 strain during the 2009 influenza virus outbreak without any modification in the Influenza Surveillance Assay (Ibis Biosciences, Carlsbad, CA, USA) [56].

Fungi are causative agents of infections, but due to the slow growth of these microorganisms, identification by culture is often impractical. Recently, a new assay for the PCR/ESI-MS systems has been validated for detection of* Aspergillus *spp.,* Candida *spp.,* Pneumocystis *spp.,* Cryptococcus *spp.,* Mucor *spp., and* Rhizopus *spp. [57]. Concordance rates between PCR/ESI-MS and phenotypic identification and sequencing were 89.7% at the genus level and 87.4% at the species level. Although most of the experiments in this study were performed with reference strains and clinical isolates, detection of* Aspergillus terreus* directly from a culture-negative bronchioalveolar lavage was demonstrated [58].

## 4. Conclusions

Microbiological diagnosis has historically relied on culture. Isolation of the causal agent provides an irrefutable proof of an infection and allows pathogen identification and determination of antibiotic susceptibility. However, many microorganisms are unculturable, fastidious, or slow-growing. Additionally, prior antimicrobial treatment negatively affects culture-based tests. In the case of bloodstream infections, lack of detection is critical. A significant percentage of blood cultures are negative despite the high likelihood of a bacterial or fungal infection [2]. Lack of culturability and the time to answer mean that many septic patients are not appropriately treated. PCR/ESI-MS is a robust technology that offers a rapid alternative for the diagnosis of bloodstream as well as other infections. Although being not currently commercially available, the new presentation of the technology has been improved in several aspects that significantly enhance sensitivity. The main advantage of this technology is that it can be used on direct patient specimens, avoiding the culture step. Using this technology as a complement to conventional methods will offer a real improvement in the management of septic and other critically ill patients (i.e., patients suffering from meningitis or fever of unknown origin). Its versatility for the detection of different kinds of microorganisms will make this technology a highly valuable tool in the clinical laboratory.

## Figures and Tables

**Table 1:** Comparison between PLEX-ID and the new version of PCR/ESI-MS.

	PLEX-ID	New version
Volume of whole blood analyzed	1.25 mL	5 mL

Samples per run of nucleic acid extraction	1–24 (24-well plate format, manual dispensation of reagents and specimens)	1–6 (ready-to-use individual reagent cartridges)

Minimum number of samples during MS analysis	6 (96-well plate)	1 (one individual 16-well strip per specimen)

Preanalytical analysis equipment	4 (mechanical lysis, magnetic nucleic acid extraction, fluid handler, and thermocycler)	3 (mechanical lysis, magnetic nucleic acid extraction, and thermocycler)

Analytic equipment	1 large instrument (desalting and MS in the same instrument)	2 bench-top instruments (separation of desalting and MS)

Time-to-result	6 h	5-6 h

ESI-MS: electrospray ionization mass spectrometry.
